# Characteristics of systematic reviews evaluating treatments for COVID-19 registered in PROSPERO

**DOI:** 10.1017/S0950268821001321

**Published:** 2021-04-23

**Authors:** Ruinian Zhang, Ya Gao, Dairong Xie, Rongna Lian, Jinhui Tian

**Affiliations:** 1 The First Clinical Medical College of Lanzhou University, Lanzhou, China; 2 Evidence-Based Medicine Center, School of Basic Medical Sciences, Lanzhou University, Lanzhou, China; 3 Key Laboratory of Evidence-based Medicine and Knowledge Translation of Gansu Province, Lanzhou University, Lanzhou, China

**Keywords:** COVID-19, outcome measures, research collaboration, systematic review, treatment

## Abstract

Characteristics and research collaboration of registered systematic reviews (SRs) on treatment modalities for coronavirus disease-2019 (COVID-19) remain unclear. This study analysed research collaboration, interventions and outcome measures in registered SRs on COVID-19 treatments and pointed out the relevant problems. PROSPERO (international prospective register of systematic reviews) was searched for SRs on COVID-19 treatments as of 2 June 2020. Excel 2016 was used for descriptive analyses of the extracted data. VOSviewer 1.6.14 software was used to generate network maps for collaborations between countries and institutions. A total of 189 SRs were included, which were registered by 301 institutions from 39 countries. China (69, 36.50%) exhibited the highest output. Cooperation between countries was not close enough. As an institution, the Chengdu University of Traditional Chinese Medicine (7, 3.70%) had the highest output. There was close cooperation between institutions. Interventions included antiviral therapy (81, 42.86%), respiratory support (16, 8.47%), circulatory support (11, 5.82%), plasma therapy for convalescent patients (11, 5.82%), immunotherapy (9, 4.76%), TCM (traditional Chinese medicine) treatment (9, 4.76%), rehabilitation treatment (5, 2.65%), anti-inflammatory treatment (16, 8.47%) and other treatments (31, 16.40%). Concerning antiviral therapy (81, 42.86%), the most commonly used antiviral agents were chloroquine/hydroxychloroquine (26, 13.76%), followed by remdesivir (12, 6.35%), lobinavir/ritonavir (11, 5.82%), favipiravir (5, 2.65%), ribavirin (5, 2.65%), interferon (5, 2.65%), abiron (4, 2.12%) and abidor (4, 2.12%). The most frequently used primary and secondary outcomes were the mortality rate (92, 48.68%) and hospital stay length (48, 25.40%), respectively. The expression of the outcomes was not standardised. Many COVID-19 SRs on treatment modalities have been registered, with a low completion rate. Although there was some collaboration between countries and institutions in the currently registered SRs on treatment modalities for COVID-19 on PROSPERO, cooperation between countries should be further enhanced. More attention should be directed towards identifying deficiencies of outcome measures, and the standardisation of results should be maximised.

## Introduction

In late December 2019, an outbreak of pneumonia of unknown origin was characterised by strong interpersonal transmission. Then scientists named the condition coronavirus disease-2019 (COVID-19) caused by severe acute respiratory syndrome coronavirus 2 (SARS-CoV-2) [1–4]. Within 3 months, it affected six continents [5, 6]. As of 20 August 2020, 22 817 751 cases have been reported, including 793 379 deaths [7]. After the outbreak, no effective treatment methods and specific medicines were available for COVID-19 [8]. Therefore, medical workers and researchers actively carried out trials to evaluate the effects of various potential drugs to find an effective drug to treat COVID-19. Previous studies have shown that some trials had research design flaws and could not be completed on time, resulting in a waste of resources [9–11]. To provide high-quality evidence to support clinical practice in preventing and treating COVID-19, researchers have also registered many systematic reviews (SRs). However, it is not clear whether several SRs are exploring the same drugs. Furthermore, well-conducted SRs and meta-analyses of randomised controlled trials (RCTs) are often considered the best way to obtain evidence for clinical practice and healthcare decisions [12–14]. Therefore, these SRs must have rigorous and standard protocols to avoid outcome reporting bias, publication bias, unplanned duplication and wasting resources during the COVID-19 pandemic [15]. However, no research has focused on the characteristics of these registered SRs.

The international prospective register of systematic reviews (PROSPERO) is an international database for prospectively registered SRs in health and social care, welfare, public health, education, crime, justice and international development. The current study evaluated the cooperation between countries and institutions and the distribution of outcome measures in registered COVID-19 treatment SRs to provide a reference for researchers to register and carry out COVID-19 SRs.

## Materials and methods

### Data sources

We systematically searched the PROSPERO registration platform (https://www.crd.york.ac.uk/prospero) to identify all the registered SRs on COVID-19 treatment. The deadline for data retrieval was 2 June 2020. We followed the search strategy proposed by the relevant section in the PROSPERO database. The search strategy is (((coronavirus OR corona-virus) AND (wuhan OR beijing OR shanghai OR Italy OR South-Korea OR korea OR China OR Chinese OR 2019-nCoV OR nCoV OR COVID-19 OR Covid19 OR SARS-CoV* OR SARSCov2 OR ncov)) OR (pneumonia AND Wuhan) OR COVID-19 OR 2019-nCoV OR SARS-CoV OR SARSCOV2 OR 2019-nCov OR ‘2019 coronavirus’ OR ‘2019 corona virus’ OR covid19 OR ncov OR ‘novel corona virus’ OR ‘new corona virus’ OR ‘nouveau corona virus’ OR ‘2019 corona virus’ OR ‘novel coronavirus’ OR ‘new coronavirus’ OR ‘nouveau coronavirus’ OR ‘2019 coronavirus’). We used the filters offered by the PROSPERO database to screen treatment SRs.

### Inclusion and exclusion criteria

Inclusion criteria: the included studies were registered SRs on PROSPERO. The study population consisted of patients diagnosed with COVID-19, with no age, gender, race and disease course restrictions. The intervention was expected to treat patients with COVID-19. The treatment methods consisted of single drug treatment, combined drug treatment, healthcare treatment and traditional Chinese medicine (TCM) treatment, etc.

We excluded SRs of animals, basic sciences, diagnostic testing, epidemiological research and health services without relevant data. Duplicate records were also excluded.

### Study selection and data extraction

Two researchers independently reviewed, screened and retrieved records based on pre-determined inclusion and exclusion criteria; they then exchanged their data with each other. Any disagreements were resolved through communication with the third researcher.

One researcher used a predefined form to extract detailed data from the included registrations, and another reviewer verified the accuracy of the extracted data. The specific data included subject, author, registration time, expected completion time, research type of included studies in SRs, discipline type, interventions, control measures, country, institution, tools used to evaluate the risk of bias in studies included in SRs, tools used to assess the certainty of the evidence, reporting or not reporting the search strategy, language and the names of databases searched, primary and secondary outcomes, software used for data analysis, and funding sources.

### Data management and analysis

We preprocessed the extracted data and standardised institutions, interventions and outcomes with different expressions. Microsoft Excel was used for the descriptive analysis of the extracted data. Then, VOSviewer 1.6.14 (Leiden University, Leiden, Netherlands) software was used to evaluate the relationship between countries and institutions and generate the corresponding cooperation network diagram, in which the nodes represent the elements of analysis (countries and institutions), node size shows the frequency, the node colours indicate different clusters and lines represent the cooperation between different nodes [16–18]. The connection between the nodes represents a co-occurrence relationship. The thicker the connection, the higher the co-occurrence frequency and correlation degree [19–21]. The VOSviewer parameters include the counting method (fractional counting), ignoring documents with multiple authors (the maximum number of authors per document was 25).

## Results

### General characteristics of registered SRs

By 2 June 2020, 205 SRs were retrieved, of which 189 met the inclusion criteria. By 3 August 2020, 122 SRs had reached the expected completion time, of which 111 SRs were still under review, with only 11 SRs completed but not yet published. All the details are presented in Table 1.

**Table 1.** Basic information

Items	*N*	Percentage (%)
Registration month		
March	25	13.23
April	109	57.67
May	50	26.46
June	5	2.65
Publication		
Review ongoing	178	94.18
Review completed, not published	11	5.82
Report the risk of bias assessment		
Yes	164	86.77
No	25	13.23
Tools used to assess the risk of bias		
Cochrane risk of bias tool	129	68.25
NOS	44	23.28
ROBINS-I	12	6.35
Whether used GRADE or not		
Yes	31	16.40
No	158	83.60
Report analysis software		
Yes	100	52.91
No	89	47.09
Data analysis software		
Review Manager	60	31.75
Stata	32	16.93
R software	13	6.88
SPSS	4	2.12
Types of included studies		
No restrictions	21	11.11
RCTs	124	65.61
Cohort studies	42	22.22
Observational studies	26	13.76
Case reports	14	7.41
Case series	22	11.64
Cross-sectional studies	4	2.12
Non-RCTs	12	6.35
Funding		
None	136	71.96
Yes	53	28.04

GRADE, grading of recommendations assessment, development and evaluation; NOS, Newcastle-Ottawa quality assessment scale; RCTs, randomised controlled trials; ROBINS-I, risk of bias in non-randomised studies - of interventions.

Of 189 included SRs, 164 (86.77%) assessed the risk of bias in the included studies, and the remaining 25 (13.23%) did not assess the quality of the studies. The most commonly used tool for the risk of bias was the Cochrane risk of bias tool (129, 68.25%), followed by the Newcastle-Ottawa quality assessment scale (NOS) (44, 23.28%), and the risk of bias in non-randomised studies - of interventions (ROBINS-I) (12, 6.35%). Only 31 (16.40%) SRs reported using grading of recommendations assessment, development and evaluation (GRADE) to assess the certainty of evidence, whereas 83.60% of SRs did appraise the certainty of evidence. In addition, 89 (47.09%) SRs did not mention the data analysis software used, and the remaining 100 (52.91%) reported it. The most widely used software was the Review Manager, with 60 (31.75%) SRs. Stata (32, 16.93%), R software (13, 6.88%) and SPSS (4, 2.12%) ranked second to fourth, respectively.

Concerning research funding sources, 136 SRs were not funded, accounting for 71.96%, and 53 SRs were funded, accounting for 28.04%. The most frequent financial assistance source was the National Natural Science Foundation of China for 11 SRs, accounting for 20.75% of all financial assistance. All the details are presented in Table 1.

The total 189 SRs were divided into four categories: conventional meta-analysis, network meta-analysis, narrative synthesis and individual patient data meta-analysis, with 152 (80.42%), 21 (11.11%), 14 (7.41%) and 2 (1.06%) SRs in each category. The research types included in these SRs were diverse. The most frequent study type was RCTs (*n* = 124, 65.61%). The other research types were relatively less frequent, including cohort studies (42, 22.22%), observational studies (26, 13.76%), case series ( 22, 11.64%), case report ( 14, 7.41%) and non-RCTs (12, 6.35%). All the details are presented in Table 1.

**Fig. 1.**
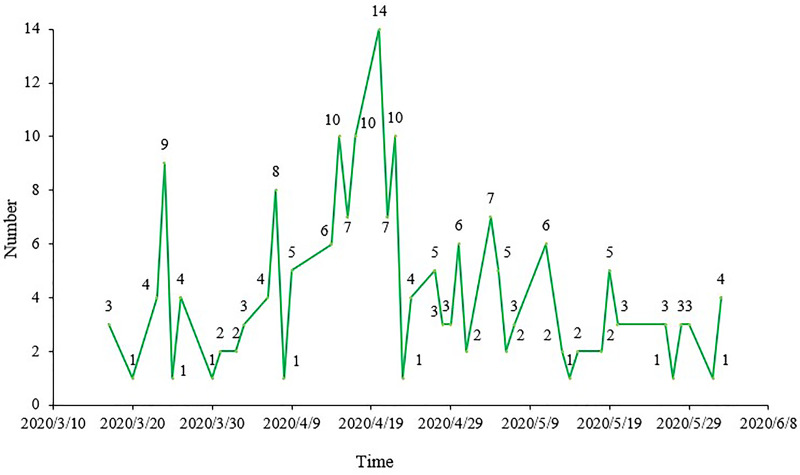
Registration time of COVID-19 treatment SRs.

Among the SRs included, 25, 109, 50 and 5 SRs were registered in March, April, May and June, respectively, accounting for 13.13%, 57.67%, 26.46% and 2.65%, respectively. Concerning specific dates, the number of registered SRs on 20 April 2020, was the largest, with 14 SRs. The relationship between the specific date and the number of registered SRs is presented in Figure 1.

### Database

Of the 189 SRs that met the standards, 188 searched the databases, and only one did not. In the reported databases, 136 (72.34%) SRs used only English databases, and the remaining 52 (27.66%) searched both English and Chinese databases. Among the databases searched, PubMed/Medline (187, 99.47%) was the most frequent one, followed by EMBASE (152, 80.85%), Cochrane Library (127, 67.54%), Web of Science (61, 32.45%), Scopus (37, 19.68%), and Google Scholar (34, 80.85%). The commonly searched Chinese databases were CNKI (49, 26.06%), Wanfang (35, 18.62%), and VIP (26, 13.83%). Commonly used database combinations were PubMed/Medline and EMBASE (152, 80.85%), PubMed/Medline combined with Cochrane Library (127, 67.55%), EMBASE and Cochrane Library (109, 57.98%) and PubMed/Medline, EMBASE, Cochrane Library, and Web of Science (36, 19.15%). The detailed database retrieval process is presented in Table 2.

**Table 2.** Reported information concerning the literature search

Category	Characteristic	*N*	Percentage (%)
Reported search strategy (*n* = 189)	Yes	188	99.47
	No	1	0.53
Language of databases searched (*n* = 188)	English	136	72.34
	English + Chinese	52	27.66
Name of database (*n* = 188)	WHO Trials	17	9.04
	Web of Science	61	32.45
	Wanfang	35	18.62
	VIP	26	13.83
	Scopus	37	19.68
	PubMed/Medline	187	99.47
	MedRxiv/BioRxiv	16	8.51
	Google Scholar	34	18.09
	EMBASE	152	80.85
	Cochrane Library	127	67.55
	CNKI	49	26.06
	ClinicalTrials.gov	26	13.83
	CINAHL	21	11.17
	CBM	29	15.43
Combination of databases (*n* = 188)	PubMed/Medline + Embase + Cochrane Library	109	57.98
	PubMed/Medline + Embase	152	80.85
	PubMed/Medline + Cochrane Library	127	67.55
	CNKI + PubMed/Medline	49	26.06
	Embase + Cochrane Library	109	57.98
	PubMed/Medline + Embase + Cochrane Library + Web of Science	36	19.15

### Country

**Fig. 2.**
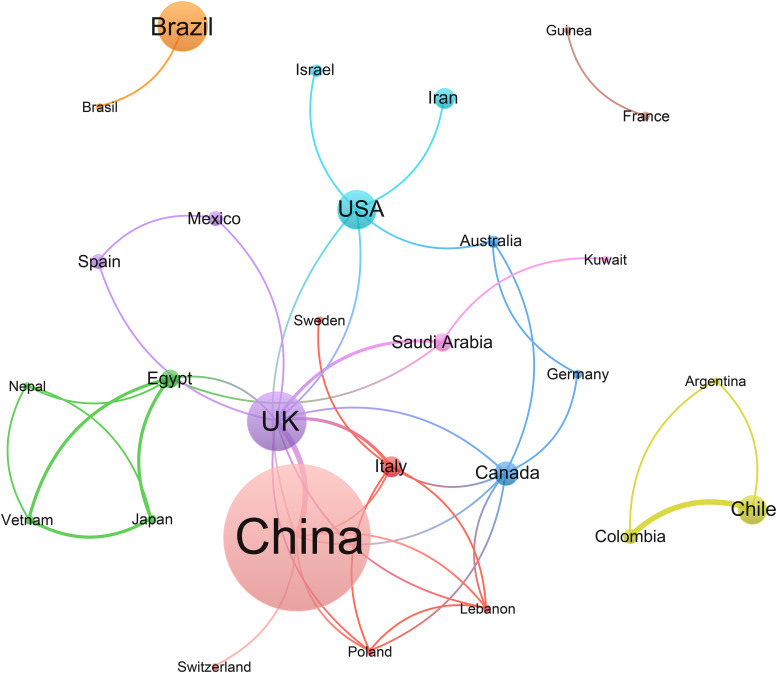
Social network analysis of countries. *Note*: Nodes represent countries; node size shows the frequency; the colour of nodes indicates different clusters and lines represent the cooperation between different countries. The line between nodes represents a cooperative relationship. The thicker the line, the higher the frequency of collaboration.

Thirty-nine countries participated in the SRs; 164 (86.77%) SRs were completed by one country, 18 (9.52%) by two countries, 1 (0.53%) by four countries, 1 (0.53%) by six countries, and 1 (0.53%) by 18 countries. The highest number of registrations was made in China (69, 36.50%), followed by the UK (22, 11.60%), Brazil (19, 10.05%), USA (13, 6.88%), Chile (9, 4.76%) and India (9, 4.76%). Canada (7, 3.70%), Iran (6, 3.17%) and Italy (6, 3.17%) also registered >5 SRs. Twenty-five countries registered more than one SR (Table 3). The social network map of the cooperative relationship among the countries was drawn. The connections between the nodes represent co-occurrence relationships. There are 27 cooperative relationships among them (Fig. 2).

**Table 3.** Countries contributing to SRs in COVID-19 treatment (>1) (*N* (%))

Rank	Country	*N* (%)	Rank	Country	*N* (%)
1	China	69 (36.50)	14	Mexico	4 (2.12)
2	UK	22 (11.60)	15	Spain	4 (2.12)
3	Brazil	19 (10.05)	16	Australia	3 (1.59)
4	USA	13 (6.88)	17	Ethiopia	3 (1.59)
5	Chile	9 (4.76)	18	Israel	3 (1.59)
6	India	9 (4.76)	19	South Korea	3 (1.59)
7	Canada	7 (3.70)	20	Denmark	2 (1.06)
8	Iran	6 (3.17)	21	France	2 (1.06)
9	Italy	6 (3.17)	22	Germany	2 (1.06)
10	Egypt	5 (2.64)	23	Japan	2 (1.06)
11	Saudi Arabia	5 (2.64)	24	Peru	2 (1.06)
12	Colombia	4 (2.12)	25	Vietnam	2 (1.06)
13	Indonesia	4 (2.12)			

### Institutions

A total of 301 institutions contributed to the registration of COVID-19 treatment SRs; 101 (53.44%) SRs were completed by one organisation, 46 (24.34%) by two organisations, 23 (12.17%) by three organisations, 6 (3.17%) by four organisations, 5 (2.65%) by five organisations, 2 (1.06%) by six organisations, 2 (1.06%) by seven organisations, 2 (1.06%) by eight organisations, and 2 (1.06%) by 11 organisations.

**Fig. 3.**
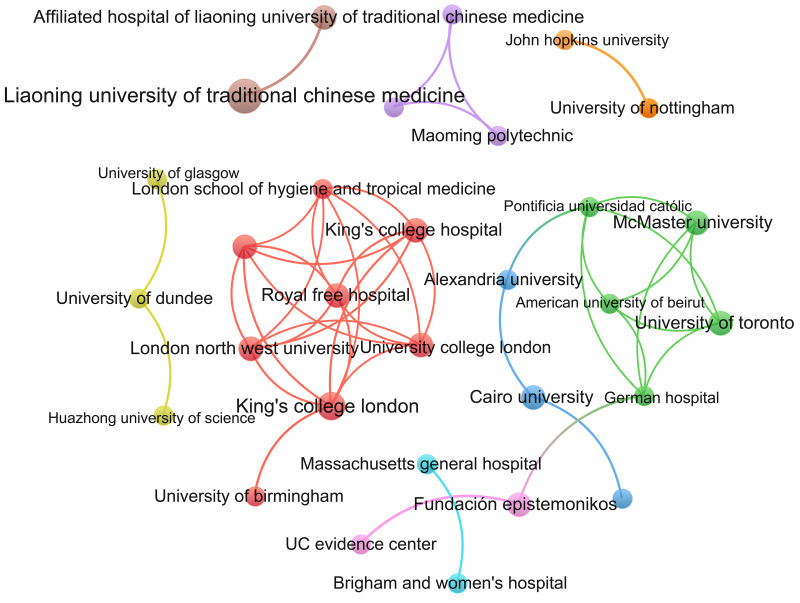
Social network analysis of institutions. *Note*: Nodes represent institutions; node size shows the frequency; the colour of nodes indicates different clusters and lines represent the cooperation between different institutions. The line between nodes represents a cooperative relationship. The thicker the line, the higher the frequency of collaboration.

The top four productive institutions are Chengdu University of Traditional Chinese Medicine (7, 3.70%), Liaoning University of Traditional Chinese Medicine (6, 3.17%), Children's Hospital of Chongqing Medical University (4, 2.12%) and King's College London (4, 2.12%) (Table 4). A social network analysis of institutions revealed that 44 institutions formed a cooperative relationship (Fig. 3).

**Table 4.** Institutions contributing to SRs on COVID-19 treatment (>2) (*N* (%))

Rank	Institution	*N* (%)
1	Chengdu University of Traditional Chinese Medicine	7 (3.70)
2	Liaoning University of Traditional Chinese Medicine	6 (3.17)
3	Children's Hospital of Chongqing Medical University	4 (2.12)
4	King's College London	4 (2.12)
5	Affiliated Hospital of Liaoning University of Traditional Chinese Medicine	3 (1.59)
6	All India Institute of Medical Sciences	3 (1.59)
7	Cairo University	3 (1.59)
8	Fundación Epistemonikos	3 (1.59)
9	King's College Hospital	3 (1.59)
10	London North West University	3 (1.59)
11	McMaster University	3 (1.59)
12	Northwick Park Hospital	3 (1.59)
13	Royal Free Hospital	3 (1.59)
14	Tehran University of Medical Sciences	3 (1.59)
15	University College London	3 (1.59)
16	University of Toronto	3 (1.59)

### Interventions

A total of 186 (98.41%) registrations reported interventions, and 3 (1.59%) did not. The reported interventions could be classified as antiviral therapy (81, 42.86%), respiratory support (16, 8.47%), circulatory support (11, 5.82%), plasma therapy for convalescent patients (11, 5.82%), immunotherapy (9, 4.76%), TCM treatment (9, 4.76%), rehabilitation treatment (5, 2.65%), anti-inflammatory treatment (16, 8.47%) and other treatments (31, 16.40%). In antiviral therapy (81, 42.86%), 11 (5.82%) registrations did not specify any specific drugs, and 70 (37.04%) indicated the drugs used. The most commonly used drugs were chloroquine/hydroxychloroquine (26, 13.76%), followed by remdesivir (12, 6.35%), lobinavir/ritonavir (11, 5.82%), favipiravir (5, 2.65%), ribavirin (5, 2.65%), interferon (5, 2.65%), abiron (4, 2.12%) and abidor (4, 2.12%) (Table 5). The control measure was placebo, conventional medicine or no treatment (189, 100%).

**Table 5.** Interventions of SRs in COVID-19 treatment

Items	*N*	Percentage (%)
Report		
Yes	186	98.41
No	3	1.59
Interventions		
Antiviral therapy	81	42.86
Not reported	11	5.82
Chloroquine/hydroxychloroquine	26	13.76
Interferon	5	2.65
Lobinavir/ritonavir	11	5.82
Ribavirin	5	2.65
Remdesivir	12	6.35
Abiron	4	2.12
Favipiravir	5	2.65
Abidor	2	1.06
Respiratory support	16	8.47
Circulatory support	11	5.82
Plasma therapy for convalescent patients	11	5.82
Immunotherapy	9	4.76
TCM treatment	9	4.76
Rehabilitation treatment	5	2.65
Anti-inflammatory treatment	16	8.47
Other treatments	31	16.40

### Outcome measures

#### Primary outcome measures

Each included SR exhibited multiple outcome indicators, with the primary outcome indicators related to symptoms, signs, examinations, prognoses, etc. The most common outcome measure was mortality rate (92, 48.68%), followed by adverse events (28, 14.81%), time of seronegativity for the coronavirus (22, 11.64%), survival rate (14, 7.41%), length of hospital stay (14, 7.41%), time to achieve clinical recovery (13, 6.88%), defervescence time (13, 6.88%) and effectiveness/effective rate (11, 5.82%). More main outcome measures are presented in Table 6.

**Table 6.** Twenty most frequent primary outcome measures (*N* (%))

Rank	Primary outcome measures	*N* (%)	Rank	Primary outcome measures	*N* (%)
1	Mortality rate	92 (48.68)	11	Disease severity scores	9 (4.76)
2	Adverse events	28 (14.81)	12	Serum cytokine levels	8 (4.23)
3	Time of becoming negative for the coronavirus	22 (11.64)	13	Lung function	8 (4.23)
4	Survival rate	14 (7.41)	14	Length of stay in ICU	8 (4.23)
5	Length of hospital stay	14 (7.41)	15	Clinical signs	7 (3.70)
6	Time to achieve clinical recovery	13 (6.88)	16	Cure rate	6 (3.17)
7	Defervescence time	13 (6.88)	17	Time of disappearance of main symptoms	6 (3.17)
8	Effectiveness/effective rate	11 (5.82)	18	Efficacy	6 (3.17)
9	Lung CT	9 (4.76)	19	Disease duration	5 (2.65)
10	Treatment safety	9 (4.76)	20	Duration of mechanical ventilation	5 (2.65)

#### Secondary outcome measures

In addition to the primary outcome measures, 127 SRs also had secondary outcome indicators, the commonly used of which consisted of the length of hospital stay (48, 25.40%), adverse events (43, 22.75%), length of stay in the intensive care unit (ICU) (30, 15.87%), mechanical ventilation (23, 12.17%), rate of viral nucleic acid turning negative (11, 5.82%), and side effects (10, 5.29%). More details are presented in Table 7.

**Table 7.** Sixteen most frequent secondary outcome measures (*N* (%))

Rank	Secondary outcome measures	*N* (%)	Rank	Secondary outcome measures	*N* (%)
1	Length of hospital stay	48 (25.40)	9	Intubation rate	7 (3.70)
2	Adverse events	43 (22.75)	10	Time to clinical improvement	6 (3.17)
3	ICU length of stay	30 (15.87)	11	Tomography imaging	6 (3.17)
4	Mechanical ventilation	23 (12.17)	12	IL-6 levels	5 (2.65)
5	Rate of viral nucleic acid turning negative	11 (5.82)	13	C-reactive protein	5 (2.65)
6	Side effects	10 (5.29)	14	Disease recovery	5 (2.65)
7	Acute respiratory distress syndrome	9 (4.76)	15	ALT	4 (2.12)
8	Adverse drug reactions	9 (4.76)	16	Renal replacement therapy	4 (2.12)

## Discussion

By 3 August 2020, 122 of the 189 SRs included should have reached the expected completion time; however, only 11 SRs were completed, with a completion rate of only 9.02%. The low completion rate might be because the data resources obtained in the early stage were insufficient, the data acquisition method was difficult, the expected completion time was too short and the difficulty of the research was estimated incorrectly. Therefore, it should be avoided in future research, and the research should be evaluated correctly from the feasibility and time perspectives. To achieve proper planning and progress, the research should be completed in the scheduled time as far as possible.

Through the reports on the retrieval database, it is not difficult to see that of the 188 SRs, and 187 SRs have used various databases to obtain the corresponding data except for one, which used a single database. Among them, PubMed/Medline combined with EMBASE was used most frequently. Besides, 72.34% of SRs used only English databases, and the remaining 27.66% used both Chinese and English databases. In this view, most of the databases are limited to English and their data are not reasonably representative, indicating that it is advisable to search multi-language databases simultaneously to make the data more representative.

Approximately 36.51% of the SRs were undertaken in China, 11.64% in the UK and 10.05% in Brazil. Thirty-nine countries have participated in the registered SRs, and 27 countries have formed cooperative relationships, with the UK having the highest cooperation level, followed by China, Canada, Egypt and Italy. A total of 301 institutions contributed to the registered SRs, with 232 establishing cooperative relationships. The Chengdu University of Traditional Chinese Medicine, Liaoning University of Traditional Chinese Medicine and Children's Hospital of Chongqing Medical University have undertaken seven, six and four projects, respectively, all in China. Cluster analysis indicated that the collaborations between institutions and between countries were not close enough. However, many SRs investigated the same interventions and reviewed similar outcomes. Therefore, researchers should enhance communication and promote extensive cooperation between countries and institutions to avoid repeated research and wasting resources. Furthermore, updating the evidence is critical, especially under the COVID-19 pandemic. Comprehensive cooperation is conducive to promoting the completion of research and timely updating of evidence, providing the latest and most comprehensive evidence in response to COVID-19 [22].

Three of the included SRs did not report intervention methods. The interventions of the remaining 98.41% SRs were manually divided into the following categories according to the treatment methods of China's COVID-19 diagnosis and treatment plan (Seven Edition) [23]: antiviral therapy, respiratory support, circulatory support, plasma therapy for convalescent patients, immunotherapy, TCM treatment, rehabilitation treatment, anti-inflammatory treatment and other treatments. On the premise of following the treatment principles of ‘disease prevention’ and ‘treatment individualised to patient, season and locality’, TCM has achieved good results in the prevention of infection, relief of symptoms, prevention of aggravation, reduction of mortality and improvements in prognosis quality [24–27]. TCM treatment involves the application of drugs according to symptoms, aetiology, pathogenesis and clinical manifestations. Commonly used drugs include Lianhua Qingwen capsule, Jinhua Qinggan granule, Reduning injection, Shufeng Jiedu Capsule, etc. In addition, acupuncture and moxibustion are more commonly used in treating COVID-19. We focused on antiviral treatment; 11 SRs did not specify the specific drugs. The most commonly used drugs were chloroquine/hydroquinone, followed by remdesivir, lobinavir/ritonavir, favipiravir, ribavirin and interference, indicating many repeated studies, with a potential waste of scientific research resources. The description of intervention measures was not standard enough. Therefore, researchers should be more careful in future research to check the registered projects to avoid repeated research. The interventions should be described in more detail and a more systematic approach.

Among all the outcome indicators, the commonly used primary outcome indicators were mortality rate, adverse events and the time of becoming seronegative for the coronavirus. The frequently used secondary outcomes consisted of the length of hospital stay, adverse events, length of stay in the ICU and mechanical ventilation. By comparing the two types of outcome indicators, the typical items were the length of hospital stay, adverse events, length of stay in the ICU, the time of becoming seronegative for the coronavirus and mechanical ventilation monitoring. However, there are some differences between the two. The primary outcome indicators are monitoring clinical symptoms and signs, while the secondary outcome indicators tend to be prognostic indicators, laboratory test data (interleukin (IL)-6, C-reactive protein, alanine aminotransferase (ALT)) and the adverse reactions mainly focus on adverse drug reactions. Therefore, the determination of outcome indicators should be complementary as far as possible to obtain more detailed and perfect information of outcome indicators and avoid any repetition.

## Conclusions

China had the highest number of registrations, and the Chengdu University of Traditional Chinese Medicine, as an institution, had the largest output. The cooperation between countries is not as close as it should be, and the cooperation between institutions is relatively close. More comprehensive and active collaborations between different institutions and regions should be fostered to enhance communication, share information and obtain more representative experimental results. Repetitive research will lead to a waste of scientific research resources. More attention should be paid to registration to avoid duplicate research. More attention should be paid to the deficiencies in interventions and outcome measures, and the outcomes should be standardised further.

## Data Availability

The data will be made available upon request.
